# The selective serotonin reuptake inhibitor sertraline causes ocular toxicity in larvae of zebrafish (*Danio rerio*)

**DOI:** 10.3389/fphys.2026.1736110

**Published:** 2026-03-27

**Authors:** Marwin Jafari, Yann Stehly, Daniela M. Pampanin

**Affiliations:** Department of Chemistry, Bioscience and Environmental Engineering, University of Stavanger, Stavanger, Norway

**Keywords:** antidepressant, aquatic toxicology, ecotoxicology, eye development and function, histology, zebrafish

## Abstract

**Introduction:**

Selective serotonin reuptake inhibitors (SSRIs) are currently the highest prescribed class of antidepressants and are frequently detected in aquatic ecosystems. They act by increasing synaptic concentrations of serotonin, a neurotransmitter which is also involved in neuronal development. A knowledge gap remains regarding the effects of SSRIs on visual function, a process heavily relying on the neuronal system.

**Methods:**

To close this knowledge gap, zebrafish (*Danio rerio*) embryos were exposed to the SSRI sertraline (SER) at sublethal concentrations of 1, 10, 100 and 1000 µg/L. Effects on the visual system were assessed at multiple levels of biological organisation in larvae exposed for 96 h, and measurements included visual function, retinal structure, and expression of genes involved in visual function and eye development.

**Results:**

Visual function was affected after exposure to 100 and 1000 µg/L of SER, while retinal structure was significantly affected after exposure to 1000 µg/L of SER. To study the molecular processes behind these effects, gene expression was assessed, pointing out dysregulation of multiple opsin genes, as well as genes involved in retinoic acid metabolism and eye development, namely *rho*, *opn1sw1*, *opn1mw1*, *rlbp1b*, *gnat2*, and *crx*. While expression of these genes was significantly downregulated at 48 hrs post fertilisation (hpf), no significant effects were detected at 96 hpf.

**Discussion:**

The present study points out the ability of SER to cause adverse effects to visual function, a key process for early life stage fish survival. However, these effects were detected at exposure concentrations exceeding the environmental one, thus not representing an environmental threat.

## Introduction

Antidepressants are a class of active pharmaceutical ingredients (APIs) that are used in the treatment of depressive disorders. As other APIs, they have been detected in surface waters in ng/L to µg/L concentrations (Mole and Brooks, 2019; Sonya et al., 2019), where they frequently end up through wastewater, either due to a lack of treatment or incomplete removal during the wastewater treatment process, and their presence is raising concerns about environmental risks to aquatic organisms (Kasprzyk-Hordern et al., 2009). The increasing evidence for bioaccumulation of APIs (Armin et al., 2014; Muir et al., 2017) and their potential to cause adverse effects in aquatic species (Sumpter et al., 2024) leads to their classification as contaminants of emerging concern, pointing out a need to improve knowledge of their effects in non-target organisms. Antidepressants are among the most widely prescribed classes of APIs, with their usage steadily growing over the last decades, and are predicted to be the pharmaceutical class seeing the largest increase in consumption (Health, N.i.o.P, 2014). Selective serotonin reuptake inhibitors (SSRIs) are currently the leading class of prescribed antidepressants, with sertraline (SER) being the most commonly used SSRI in recent years, reaching 39.9 million prescriptions in 2022 (Kane, 2024).

SER has been measured in wastewater treatment plant effluent at concentrations up to 5.1 µg/L, via liquid chromatography tandem mass spectrometry, and in untreated sewage at concentrations up to 17 µg/L, via high-performance liquid chromatography using an UV detector (Styrishave et al., 2011; Sonya et al., 2019). Evidence for bioaccumulation of SER has been reported in fish (Kateřina et al., 2020), aquatic plants (Drobniewska et al., 2024) and marine invertebrates (Silva et al., 2017). The acute toxicity of SER is known to be relatively low, and while no LC_50_ data have yet been published for zebrafish (*Danio rerio*), studies report no significant mortality at concentrations of 1 mg/L for zebrafish embryos at 80 hours post fertilisation (hpf) (Sílvia et al., 2015). Although acute effects have been observed only at relatively high concentrations exceeding those typically found in the environment, significant behavioural effects have been reported in zebrafish larvae after exposure to SER at environmentally relevant concentrations. Behavioural changes due to SER exposure have been observed in zebrafish, including a decrease in basal locomotor activity (Faria et al., 2022), and in photomotor response, measured as locomotion after a visual stimulus from changing light conditions (Suryanto et al., 2021). Transcriptomic effects to on genes involved in eye development have also been reported in 144 hpf zebrafish exposed to 0.1 µg/L SER (Sehonova et al., 2019). Despite these molecular-level indications of potential developmental impact, no studies have yet clarified how such transcriptomic changes translate into functional outcomes towards vision. Understanding whether SER affects the visual performance of early life-stage zebrafish is crucial, as vision plays a key role in survival behaviours such as prey capture, predator avoidance, and navigation (Hollis et al., 2018).

SSRIs act by increasing the synaptic concentration of the neurotransmitter serotonin, and published studies attributed the behavioural changes caused by antidepressants solely to alterations in neurotransmitter levels (Faria et al., 2022), leading to modified behaviour. While studies detected effects to visually mediated behaviour, such as photomotor response (Suryanto et al., 2021), no known study has directly assessed the effects of SSRIs to visual function directly. As zebrafish are known to rely on vision to navigate their surrounding (Hollis et al., 2018), effects to vision would offer an alternative explanation to behavioural effects which previously were solely attributed to altered neurotransmitter levels. Serotonin is also involved in the development of the neuronal system, which vision heavily depends on (Gaspar et al., 2003; Sodhi and Sanders-Bush, 2004), as the retina is considered a part of the central nervous system, and relies on multiple types of neurons to create, process and transduce visual stimuli (Malicki et al., 2016). A recent finding from our research group has demonstrated that tricyclic antidepressants (TCAs) can adversely impact visual function and eye development in zebrafish embryos, which may in turn contribute to the observed behavioural outcomes (Jafari et al., 2026). As previously stated, vision plays an important role in the survival of adult and larval fish due to its involvement in guiding processes such as feeding and the evasion of predators (Land, 2009; Hollis et al., 2018). Thus, any impact on the visual system can be considered ecologically relevant and requires careful study. To study the effects of SSRI exposure to the visual system, early, non-protected life-stages of zebrafish, which depict a common model species in (eco-)toxicology including regulatory testing (OECD, 2013), were exposed to the SSRI SER. Endpoints at multiple levels of biological organisation were assessed in order to establish a better understanding on how antidepressants affect the visual system at molecular, structural and functional levels. By combining the optokinetic response (OKR) assay, a behavioural test for the assessment of visual function, with histological evaluation of retinal structure and analysis of gene expression related to visual function, the present study aims to address the critical knowledge gap regarding whether exposure to SER affects the visual system in early life-stage zebrafish.

## Materials and methods

Adult zebrafish of the wild-type WestAquarium strain were provided by the Aquatic Ecology and Toxicology Group at the Centre for Organismal Studies (University of Heidelberg) and kept in 4.5 L polycarbonate tanks in a LAbREED Zebrafish System (IWAKI Aquatic, USA) at the University of Stavanger research facility. The zebrafish laboratory at the University of Stavanger is an approved facility by the Norwegian Food Safety Authority (Mattilsynet), application number VSID3184 (22/09/2021), with no separate application required for the use of zebrafish embryos and larvae up to 120 hpf according to local guidelines. Artificial water was prepared by treating tap water with reverse osmosis and adding synthetic sea salt (47.5% Cl^-^, 26.3% Na^+^, 6.6% SO4^2-^, and 3.2% Mg^2+^, Instant Ocean, Aquarium Systems, Sarrebourg, France) to a conductivity of 500 µS/cm, and sodium bicarbonate (CAS 144-55-8, Iwaki Systems, USA) to a pH of 7.65. The water temperature was kept at 28 ± 1 °C and a 14:10h light:dark cycle was maintained. Fish were fed twice a day, once with GEMMA Micro 300 µm dry food (Skretting, Netherlands) equivalent to approximately 5% of their body mass, and the other time with *Artemia salina* nauplii. Three male and three female adults were bred by placing them in a Sloping Breeding Tank (Tecniplast, Buguggiate, Italy) overnight, and eggs were collected in the morning, rinsed, and examined under a light microscope to select only fertilised eggs in the early blastula stage.

SER hydrochloride (CAS 79559-97-0, Thermo Fisher Scientific, Waltham, USA) stock solutions were prepared using dimethyl sulfoxide (DMSO, CAS 67-68-5, ≥ 99.5% purity, Lab-Scan analytical sciences, Poland), and exposure solutions at a final concentration of 0.01% DMSO were prepared by dilution of stock solutions in artificial water. The pH was measured in the highest treatment concentration to exclude the possibility of acidification of the water as a factor contributing to the observed effects (FiveEasy pH meter, Mettler Toledo, Gießen, Germany).

### Exposure and experimental design

Zebrafish embryos were exposed within the first 3 hpf. The experimental design included: a negative control group containing only artificial water, a solvent control group containing 0.01% DMSO, and four SER exposure groups (1, 10, 100 and 1000 µg/L), all depicting sublethal concentrations. Exposures were carried out in 6-well polystyrene plates (VWR, US) at 28 ± 1 °C with a 14:10h light:dark cycle. The increased temperature of 28 °C compared to the 26 °C suggested to the OECD guidelines for the Fish Embryo Acute Toxicity Test (OECD, 2013) was selected as an accelerated development due to the higher temperature (Urushibata et al., 2021) led to an earlier onset of the optokinetic response (OKR) reflex, allowing for testing the visual function via the OKR assay at 96 hpf. Lethality was recorded every 24 h, dead embryos were discarded and 80% of the test solution was renewed until termination of the experiments at 96 hpf.

### Optokinetic response assay

The OKR assay was used as a behavioural assay for the assessment of the visual function of zebrafish larvae (Brockerhoff et al., 1995). At 96 hpf, larvae were immobilised in 4% methyl cellulose within 35 mm Petri dishes and positioned with the tails pointing towards the centre and the heads towards the outside of the dish. Petri dishes, containing 4 larvae each, were then placed within a cylinder displaying a pattern of alternating black and white stripes that were 9° wide, which was rotated by an attached motor at 6 revolutions per min. Eye movements were recorded at 30 frames per s via a camera (Excelis HD, ACCU SCOPE, NY, USA) positioned over the cylinder. The number of eye saccades each larva displayed over 60 s was assessed for both clockwise and counterclockwise directions, and the obtained mean value of both directions was used as the endpoint for each larva. Per exposure group, 3 replicates (n=3) each consisting out of 6 larvae were analysed.

### Histological examination

At 96 hpf, zebrafish larvae were prepared for histological examination of the retina, according to the method used by Brotzmann et al (Brotzmann et al., 2022). Briefly, larvae were euthanised via hypothermia, fixed in Davidson’s solution (9% formaldehyde, 12% glacial acetic acid, 31% ethanol, v/v) for 24 h and embedded in an 1% agarose gel. Twelve larvae per group were embedded per gel and gels were dehydrated and then infiltrated with paraffin at the Stavanger University Hospital.

Sections with a thickness of 4 µm were mounted on Superfrost Plus (Menzel, Braunschweig, Germany) slides and scanned under 40x magnification with a NanoZoomer S60 (Hamamatsu, Hamamatsu City, Japan). QuPath software (v0.4.3) was used for analysis. The diameter of the retinal pigment epithelium (RPE) was measured as the main endpoint for developmental effects to the eye, due to the implied involvement of altered retinal structure in adverse effects to visual function (Gölz et al., 2022). RPE and eye diameters were measured on the section containing the optic nerve to standardise the analysis (Baumann et al., 2015). The eyes of 8 larvae were analysed for the following groups: solvent control, 10 and 1000 µg/L SER exposure groups. Due to the loss of some sample sections containing the optic nerve, the eyes of only 7 larvae were analysed for the 1 and 100 µg/L SER exposure groups.

### Gene expression

As the OKR assay detected effects of SER in concentrations as low as 100 µg/L, qPCR was used to measure changes in expression of nine vision related genes at that exposure concentration. Gene expression was evaluated both at the 96 hpf timepoint, at which behavioural effects were detected, and at an earlier timepoint (48 h) to detect possible disruptions during early ocular development. Three replicates per group, each one consisting of 15 embryos, were used. Individuals were euthanised via hypothermia and flash frozen in liquid nitrogen. Samples were stored at -80 °C until RNA extraction. A guanidinium thiocyanate and phenol mixture (QIAzol, Qiagen, Hilden, Germany) was added to each sample and incubated for 5 min. Samples and QIAzol were transferred into sterile homogenisation tubes (Fastprep, MP Biomedicals, Irvine, USA), containing ceramic beads (Lysing Matrix D, MP Biomedicals, Irvine, USA), and homogenised via mechanical homogenisation at 5 m/s for 40s using a FastPrep-24 homogeniser (MP Biomedicals, Irvine, USA) at 5 m/s for 40s. RNA was extracted following the QIAzol protocol, and then transferred to a RNeasy Mini Spin Column for additional wash steps, according to the RNeasy protocol (Qiagen, Hilden, Germany).

RNA with absorbance ratios A260/A230 of above 1.8 and A260/A280 of above 2 were used for cDNA synthesis via the iScript™ cDNA Synthesis Kit (Bio-Rad Laboratories, Hercules, CA, USA), according to protocol with 1 µg total RNA per reaction. qPCR was run using a standardised protocol based on the SsoAdvanced Universal SYBR Green Supermix (Bio-Rad Laboratories, Hercules, CA, USA), using 100 ng of cDNA template per reaction and forward and reverse primers in concentrations of 500 nM, and a CFX Opus 96 Real-Time PCR System (Bio-Rad Laboratories, Hercules, CA, USA). For each sample, three technical replicates per primer pair were run, and for each primer pair, a no-template control was included. The PCR program consisted of an initial denaturation at 95 °C for 3 min and 40 cycles of denaturation at 95 °C for 10 s, annealing and extension at 60 °C. Specificity of the primers was confirmed via generating melt curves of the qPCR product. Fold changes against the solvent control were calculated via the 2^-ΔΔCt method using β-actin as a reference gene (Schmittgen and Livak, 2008). The primer sequences are listed in **Supplementary Table 1**.

### Statistical analysis

The statistical analysis was performed using R v4.2.2 (R Core Team, 2022). Shapiro-Wilk test and QQ-plots were used to test for normal distribution, and Levene’s test was used to test for homogeneity of variance. A Welch ANOVA with a Tukey’s range *post hoc* test was used for the OKR assay and eye histology data. For the gene expression data, Welch’s t-test with Benjamini-Hochberg correction for multiple observations was used. Results with a p-value < 0.05 when compared to the solvent control were considered statistically significant. The results of the OKR assay were depicted as a boxplot to visualise the variance of the data, since behavioural endpoints commonly depict high variability (Fischer et al., 2025).

## Results

### Optokinetic response assay

No significant mortality occurred in any of the exposure groups (**Supplementary Table 2**). The mean eye saccades in the control and solvent control groups at 96 hpf were 10.9 ± 3.01 and 10.9 ± 3.36 saccades/min, respectively, with no significant difference between these two groups. For the 1 and 10 µg/L SER exposure groups, no significant effects were detected when eye saccades were compared to the solvent control group data. After exposure to 100 and 1000 µg/L SER, significant decreases of 34 and 86% in eye movement were detected in comparison to the solvent control group, with means of 7.17 ± 3.18 and 1.53 ± 1.64 saccades/min, respectively.

### Histological examination

**Figure 1:**
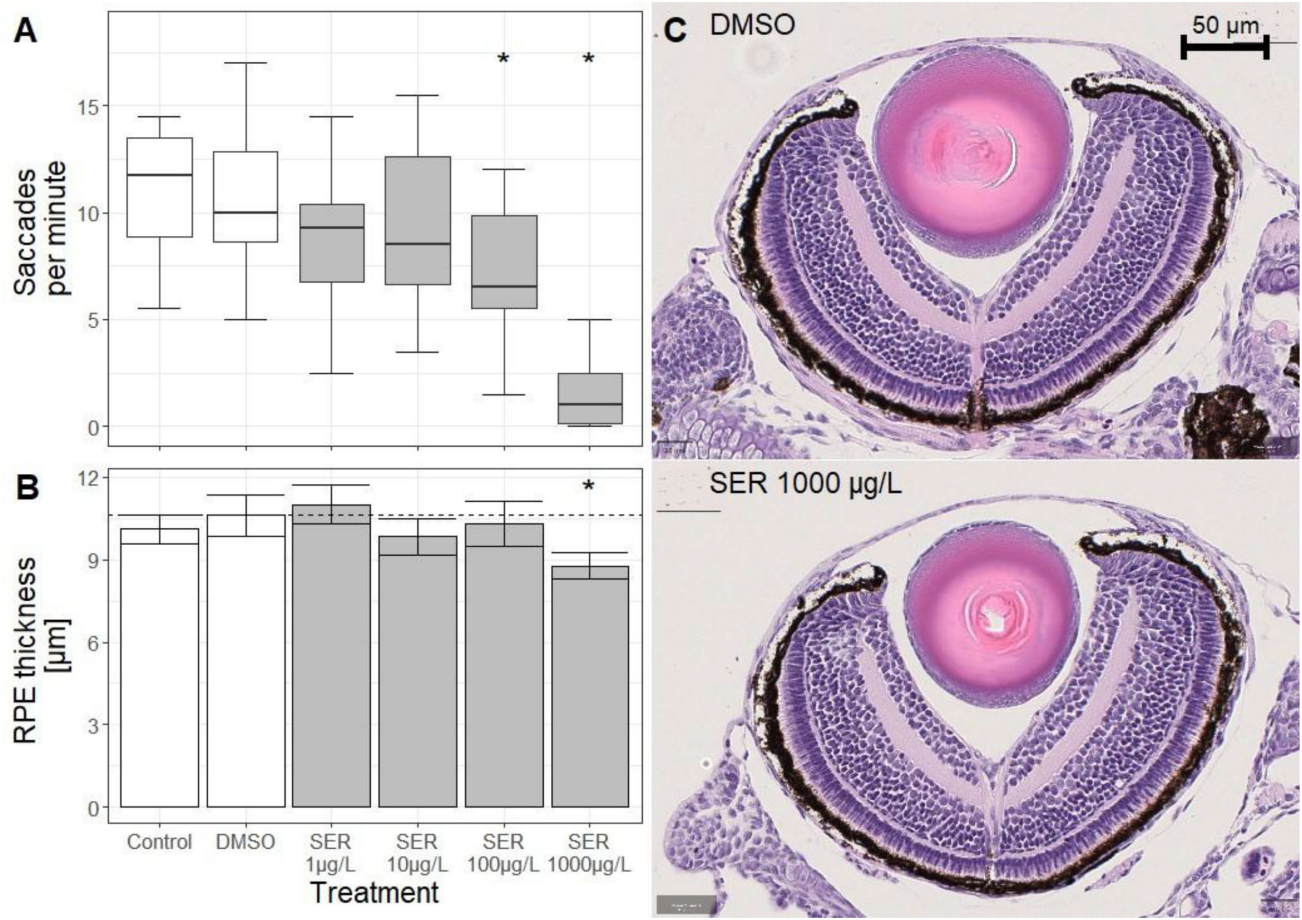
Effects of sertraline (SER) to eye structure and function. **(A)** Median number of eye saccades per minute in zebrafish (Danio rerio) larvae at 96 hours post fertilisation (hpf). The upper and lower limits of the box depict the 25th and 75th percentile, and the whiskers depict maximum and minimum values. **(B)** Mean retinal pigment epithelium (RPE) thickness of zebrafish larvae (96 hpf). Dotted line depicts the mean eye diameter of the solvent control. Error bars depict standard deviation. **(C)** Representative images of 96 hpf larval eyes stained with haematoxylin and eosin (40x magnification) in the solvent control group (DMSO). Asterisks (*) denote significant difference (p < 0.05) compared to the solvent control, according to Welch ANOVA.

No significant effects in RPE thickness nor eye diameter were observed between larvae in the negative and solvent control group (**Figure 1**), with mean RPE thickness of 10.11 ± 0.53 and 10.61 ± 0.75µm, and a mean eye diameter of 276.5 ± 13.0 µm and 276.8 ± 9.0 µm, respectively. A significant decrease of RPE thickness compared to the solvent control was recorded after exposure to 1000 µg/L SER (8.77 ± 0.48 µm, 13% decrease). No significant effects related to both the RPE thickness and the eye diameter were recorded in the other exposure groups, compared to the solvent control group.

### Gene expression

**Figure 2:**
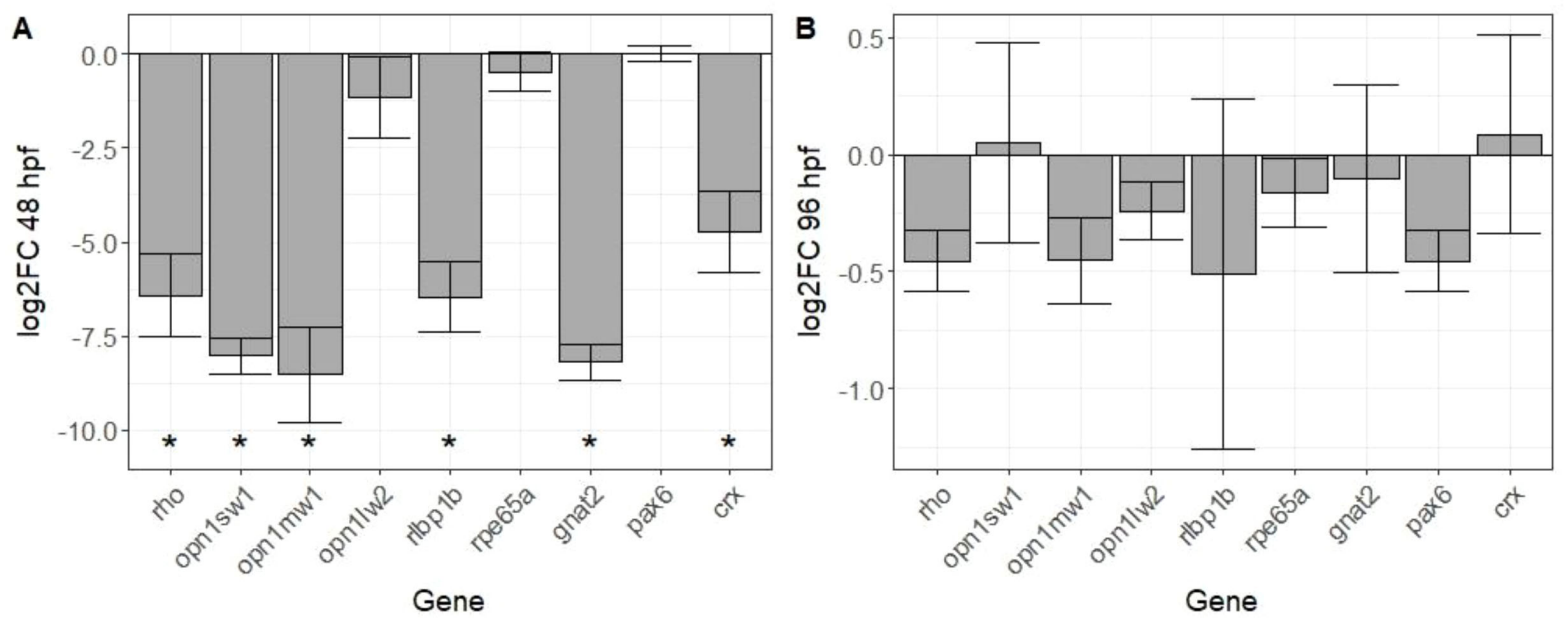
Mean relative expression of nine genes related to visual system development after exposure to 100 µg/L sertraline in **(A)** zebrafish embryos (48 hpf) and **(B)** zebrafish larvae (96 hpf). Expression levels are reported as log2-transformed fold-changes relative to the solvent control and were determined using the Δct method with β-actin as a reference gene. Error bars depict standard error. Asterisks (*) denote significant difference (p < 0.05) of relative expression levels to solvent control (Welch’s t-test with Benjamini-Hochberg correction).

The expression of nine genes playing a key role in vision and the development of the visual system was analysed in both 48 hpf and 96 hpf zebrafish after exposure to 100 µg/L SER (**Figure 2**). At 48 hpf, the expression of the opsin genes (*rho*, *opn1sw1* and *opn1mw1*) was significantly decreased in the 100 µg/L SER exposure groups, as well as *rlbp1b*, which is involved in retinoic acid transport, *gnat2*, which is involved in visual signal transduction, and *crx*, a transcription factor involved in the development of photoreceptors. At 96 hpf, no significant effects on any of the selected genes were detected when comparing data from the exposed group and the solvent control.

## Discussion

Previous studies have assessed the transcriptomic effects of SER on zebrafish, with some of the results pointing towards adverse effects to the visual system. For example, enrichment analysis after exposure of juvenile zebrafish to 100 µg/L SER revealed affected pathways such as rhodopsin signalling (Huiting et al., 2022), and 168 hpf larval zebrafish exposed to 25 and 250 µg/L SER revealed significant dysregulation of genes associated with vision and eye development, such as *opn1mw2* (Park et al., 2012). *pax6*, which codes for a transcription factor involved in eye development, was also found to be dysregulated after exposure to 0.1 and 10 µg/L SER in 144 hpf zebrafish larvae (Sehonova et al., 2019). While these previous studies focused on developmental timepoints after 120 hpf, which can be considered animal experiments due to the onset of independent feeding in zebrafish embryos at 120 hpf (Achenbach et al., 2020), this research found evidence for ocular toxicity of SER, including transcriptomic effects, in early, non-protected life stages of zebrafish.

This is the first known study finding evidence for ocular toxicity of an SSRI to zebrafish embryos. Significant decreases in visual function were detected after exposure to 100 and 1000 µg/L via the OKR assay, which quantifies eye movements in response to a visual stimulus. Previous studies have shown behavioural effects in response to visual stimuli using a photomotor response assay for concentrations from 1 µg/L SER (Suryanto et al., 2021), which measures locomotion in response to a light pulse. However, while including a visual stimulus, are generally used to assess behavioural effects of compounds acting on the neuronal system and are not well linked to visual function (Ortmann et al., 2022), in contrast to the OKR, which depicts an endpoint directly associated with visual function (Brockerhoff et al., 1995) that has successfully been implemented to assess ocular toxicity of chemicals (Baumann et al., 2015; Magnuson et al., 2020). Thus, while effects on the visual function might contribute to the photomotor response behaviour, this study is the first known study assessing effects of SER to visual function in fish. Antidepressants, including SSRIs, have shown the ability to affect the behaviour of non-target organisms through the modulation of neurotransmitter levels (Fonseka et al., 2016), and in here, the results of the OKR could also be explained with such behavioural effects. However, the effects of SER exposure on the eye structure, determined via the histological assessment of the retina, support the conclusion that SER directly affects the visual system of larval zebrafish. While these histological effects were only observed after exposure to 1 mg/L of SER, the dysregulation of genes which play an underlying role in vision after exposure to 100 µg/L SER can be directly linked to the decrease in visual function observed in the same exposure group. Effects on expression of genes involved in vision were only detected at the 48 hpf timepoint, not at the 96 hpf, which is line with the findings from a previous study assessing transcriptomic responses of zebrafish to TCAs during early embryonic development (Jafari et al., 2026). Effects on *crx* can be linked to impairment of the retinal structure and photoreception due to the involvement of the gene in retinogenesis and the differentiation of photoreceptors (Shen and Raymond, 2004). Dysregulation of genes for both rod and cone opsins (*rho*, *opn1sw1* and *opn1mw1*) can also directly be linked to adverse visual function, as they code for photoreceptor molecules essential for visual perception (Vihtelic et al., 1999), and suggests that photoreceptors might directly be affected. While these transcriptomic effects preceded the timepoint at which effects on visual function and retinal structure were detected, the dysregulation of genes during critical stages in early embryonic development can still contribute to effects on the visual system at later life stages. A potential reason why significant effects to gene expression were only detected at the 48 hpf timepoint and not at 96 hpf could be a higher sensitivity of earlier life-stages to specific xenobiotics. In a previous study assessing transcriptomic effects of tricyclic antidepressants to zebrafish embryos at 48, 72 and 96 hpf, the highest number of differentially expressed genes was reported at 48 hpf, with the number of differentially expressed genes decreasing over time (Jafari et al., 2026). The absence of effects at the 96 hpf timepoint could also point out an adaptation of the neuronal system to antidepressants, and while further studies are required to confirm this to occur in zebrafish, a decrease of antidepressant efficacy over treatment time mediated by habituation of the neuronal system has been reported in patients undergoing treatment (Fava, 2003). Exposure to 0.1 and 1 µg/L of SER was reported to cause a significant downregulation of the transcription factor gene *pax6* in 144 hpf zebrafish larvae (Sehonova et al., 2019), however this study found no significant effects on the expression of *pax6*, neither at 48 nor 96 hpf. This can either be explained with transcriptomic effects to genes commonly not following monotonic dose-response relationships (Larras et al., 2018), or to difference in the sensitivity of that gene’s expression to stressors between these timepoints. Other studies assessing transcriptomic effects in zebrafish also found effects to genes and pathways involved in vision (Sehonova et al., 2019; Huiting et al., 2022), however this is the first study that studied effects of SER on gene expression in non-protected early life stages of zebrafish.

Exposure to SSRIs including SER have been demonstrated to affect serotonin levels and cause behavioural alterations in zebrafish (Faria et al., 2022), but neither SSRI exposure nor serotonin dysregulation have been linked to visual function before. While clinical studies are available reporting maculopathy, a pathological condition of the retina, in connection with SER treatment (Mason and Patel, 2015; Javidi et al., 2021), this is the first study reporting adverse effects on visual function in fish after exposure to SER. Altered retinal structure has been linked to effects on visual function in zebrafish larvae exposed to substances interfering with thyroid signalling (Gölz et al., 2022), and while this study observed effects at both of these endpoints, only visual function was affected in the 100 µg/L SER exposure group. This implies that in the case of SER, effects on visual function are not caused by effects on retinal structure, as decreased visual function was detected in concentrations where no altered retinal structure was observed, and they rather might share the same underlying causative mechanism.

The role of serotonin in the development of the neuronal system could offer an explanation on how exposure to SER might lead to ocular toxicity in zebrafish larvae. Serotonin is known to modulate processes such as neurogenesis and neuroplasticity (Gaspar et al., 2003) and is involved in the development of the central nervous system (Daubert and Condron, 2010). With the retina being part of the central nervous system and multiple neuron types playing a role in vision, effects on neuronal development could offer a possible mechanism through which SER might cause effects to the visual function. Serotonin receptors which are conserved in zebrafish (de Moura et al., 2023) have been shown to be required for the retinal processing of visual information in murine models (Trakhtenberg et al., 2017), suggesting serotonin to be directly involved in the vision. Other neurotransmitters such as dopamine and gamma-aminobutyric acid have also been shown to be involved in neuronal signalling in the retina (Hirasawa et al., 2015). Although they are not the direct targets of SSRIs such as SER, dopamine metabolites have been found to be upregulated in zebrafish larvae after exposure to SER (Faria et al., 2022). A decrease in thyroid hormone levels, specifically T4, has also been observed in patients undergoing treatment with SSRIs, including SER (Eker et al., 2008; Liao et al., 2023), and rats showed a similar response after administration of serotonin and SER (Sullo et al., 2011; Azimzadeh et al., 2015). Since decreased thyroid hormone levels have been linked to decreased visual function and RPE thickness in zebrafish larvae (Baumann et al., 2015; Gölz et al., 2022), future studies should focus on elucidating the effects of antidepressants on thyroid signalling in fish, which could contribute to elucidating their effects on the visual system. While the mechanism through which SER exposure caused the adverse effects towards the visual system reported in this study is still unknown, mechanisms are reported in the literature that offer possible links between the known mechanism of SER, namely the modulation of serotonin levels, and ocular toxicity. Future studies should investigate the role that neuroendocrine or thyroid disruption and developmental neurotoxicity play to further clarify the mechanism of action behind the ocular toxicity of antidepressants.

This study found evidence for ocular toxicity of SER to zebrafish embryos. A previous study has reported ocular toxicity of the TCAs amitriptyline and nortriptyline (Jafari et al., 2026). However, as TCAs show lover specificity towards the serotonin transporter, and higher side effect burden compared to SSRIs (Anderson, 2000; Arias et al., 2018), it was unclear if the effects occur downstream of modulated serotonin levels and would extend to other classes of antidepressants. Thus, the present study is the first one showing evidence of ocular toxicity in fish, via both visual function and eye structure, as an adverse effect of exposure to an SSRI, which depicts the most used class of antidepressants, with sertraline specifically being the most prescribed SSRI (Chu and Wadhwa, 2024; Kane, 2024). While ocular toxicity depicts an ecologically relevant adverse endpoint contributing to survival of fish larvae (Blaxter, 1986), effects of SER on the visual system of zebrafish larvae were only observed at concentrations exceeding concentrations detected in the environment. This study found effects to the visual system only after exposure to 100 and 1000 µg/L SER, which depicts concentrations exceeding those detected in the environment, as the SSRI is most frequently detected in the environment in the ng/L range (Kostich et al., 2014; Sonya et al., 2019), with the highest environmental concentration reported being 17 µg/L (Styrishave et al., 2011).

## Conclusion

The present study shows evidence that the SSRI SER, a commonly used pharmaceutical in the treatment of depressive disorders which is frequently detected in the aquatic environment, causes ocular toxicity in zebrafish embryos. Exposure to SER was shown to decrease visual function and alter retinal structure as well as expression of genes involved in vision., although no effects were observed at environmentally relevant concentrations. While the findings of this study suggest no environmental risk of SER under current environmental conditions, the multitude of antidepressants acting via similar mechanisms found in the environment, as well as the predicted increase of antidepressant consumption, might lead to potential risks from additive effects and increasing environmental concentrations. Further research is needed to determine the mechanism through which SER affects the visual system. The present study also points out the usefulness of zebrafish embryos as an alternative model to animal testing for studies assessing ocular toxicity.

## Data Availability

The original contributions presented in the study are included in the article/**Supplementary Material**. Further inquiries can be directed to the corresponding author.

## Supplementary material

Supplementary Table 1 Sequences of primer pairs used for gene expression analysis via qPCR.

Supplementary Table 2 Mortality across exposure groups.
