# Supplementary material for: The selective serotonin reuptake inhibitor sertraline causes ocular toxicity in larvae of zebrafish (Danio rerio)
Source: Front Physiol. 2026 Mar 27;17:1736110. doi: 10.3389/fphys.2026.1736110 (PMC13066282; doi:10.3389/fphys.2026.1736110)
Supplement: Supplementary Table 1 — Sequences of primer pairs used for gene expression analysis via qPCR. [file Table1.docx]

Table S1. Sequences of primer pairs used for gene expression analysis via qPCR.

| Gene name | Primer sequences (5’-3’) | Reference |
| --- | --- | --- |
| *β-actin* | Forward: AAGAGCTATGAGCTGCCTGA  Reverse: ACCGCAAGATTCCATACCCA | (Gonzalez et al., 2006) |
| *opn1sw1* | Forward: TTCCAAAGTCAGCCCCTTCG  Reverse: GTTCATAGGTGTGCCCACGA | (Laranjeiro & Whitmore, 2014) |
| *opn1mw1* | Forward: ACACCCTTTTCTGTGGCAAG Reverse: ATGACGGAGCACTGAATAGGC | (Laranjeiro & Whitmore, 2014) |
| *opn1lw2* | Forward: CACAATCAGCGTCATCAATC  Reverse: AGTCCAGCAATACCACATAC | (Houbrechts et al., 2016) |
| *rho* | Forward: ACTTCCGTTTCGGGGAGAAC  Reverse: GAAGGACTCGTTGTTGACAC | (Laranjeiro & Whitmore, 2014) |
| *rpe65a* | Forward: GCCCCGCAGCCAGAAGTCAG  Reverse: ACGAGTCGGGCTCCTGCCAT | (Higdon et al., 2013) |
| *rlbp1b* | Forward: TGAGACGGATGAGAAGCGAA  Reverse: ACCTCACAAGCACGCCAT | Generated via Primer BLAST |
| *pax6* | Forward: CCACACCGTACGGGATTCAA  Reverse: TCCCAGCGTCCCTCTTATCT | Generated via Primer BLAST |
| *gnat2* | Forward: CGTGATCTGAGGTACAGGGC  Reverse: GCTACCCATCTCGTCGTCTG | (Laranjeiro & Whitmore, 2014) |
| *crx* | Forward: ATCCACTGTGTGGTTCAGGC  Reverse: GCTGTAGGAAGAGGGCTGAC | (Laranjeiro & Whitmore, 2014) |

References

Gonzalez, P., Baudrimont, M., Boudou, A., & Bourdineaud, J. P. (2006). Comparative effects of direct cadmium contamination on gene expression in gills, liver, skeletal muscles and brain of the zebrafish (Danio rerio). *Biometals*, *19*(3), 225-235. <https://doi.org/10.1007/s10534-005-5670-x>

Higdon, C. W., Mitra, R. D., & Johnson, S. L. (2013). Gene Expression Analysis of Zebrafish Melanocytes, Iridophores, and Retinal Pigmented Epithelium Reveals Indicators of Biological Function and Developmental Origin. *PLOS ONE*, *8*(7), e67801. <https://doi.org/10.1371/journal.pone.0067801>

Houbrechts, A. M., Vergauwen, L., Bagci, E., Van houcke, J., Heijlen, M., Kulemeka, B., Hyde, D. R., Knapen, D., & Darras, V. M. (2016). Deiodinase knockdown affects zebrafish eye development at the level of gene expression, morphology and function. *Molecular and Cellular Endocrinology*, *424*, 81-93. <https://doi.org/https://doi.org/10.1016/j.mce.2016.01.018>

Laranjeiro, R., & Whitmore, D. (2014). Transcription factors involved in retinogenesis are co-opted by the circadian clock following photoreceptor differentiation. *Development*, *141*(13), 2644-2656. <https://doi.org/10.1242/dev.104380>
