# Supplementary material for: The selective serotonin reuptake inhibitor sertraline causes ocular toxicity in larvae of zebrafish (Danio rerio)
Source: Front Physiol. 2026 Mar 27;17:1736110. doi: 10.3389/fphys.2026.1736110 (PMC13066282; doi:10.3389/fphys.2026.1736110)
Supplement: Supplementary Table 2 — Mortality across exposure groups. [file Table2.docx]

Table S2. Mortality across exposure groups

| Exposure group | Control | DMSO | SER  1 µg/L | SER  10 µg/L | SER  100 µg/L | SER  1000 µg/L |
| --- | --- | --- | --- | --- | --- | --- |
| Mortality [%] | 2.1 | 2.2 | 1.0 | 0.8 | 2.4 | 1.2 |
